# Socio-demographic profile of HIV/AIDS patients at ART centres in Chennai

**DOI:** 10.1186/1471-2334-12-S1-P53

**Published:** 2012-05-04

**Authors:** T Uma, Parameswari Srijayanth, S Valarmathi, S Sekar, N Kabilan, Mayilvahanan Natarajan

**Affiliations:** 1 Department of Epidemiology, The Tamilnadu Dr. MGR Medical University, India; 2 Senior Medical Officer, ART centre, Madras Medical College, Chennai, India

## Background

To study the socio-demographic status of HIV patients in ART centres, Chennai, Tamilnadu.

## Methods

After obtaining informed consent, a semi-structured questionnaire was administered to adult HIV patients attending ART centers, Chennai. Their demographic details, personal history, behavioral pattern were studied between June-October 2011.

## Results

Out of 296 patients, 117 were male, 174 were female and 5 were transgender. 77.4% of them were between the age group of 18-41. Among all, 20.6% were illiterate and 79.4% were literate. Among the literates, 5.7% were graduates. In men, 77.8% were married. Among married males 5.1% were separated and 7.7% were widowers. In women, 97.7% were married. Among them 29.9% were widow and 9.8% were separated. 84.4% of male and 28.1% of female were employed. 56.6% were daily wagers. 57.7% were earning <4000rs per month. Livelihood of 71.9% females was supported by others, 32.2% were housewives. 57% of patients were in a nuclear family and 7.4% of them were in broken family. 67.5% of the male have at least any one of habits like alcohol, smoking and intravenous drug usage. 71% males and 15.6% females had multiple sexual partners. Among males, 10.25% were homosexuals and 1.7% was heterosexuals. 53.9% of patients use safe drinking water.

## Conclusion

Low socio-economic status with high risk behaviour and lack of awareness were prevailing among the HIV patients. Epidemiological studies should be carried out in various settings to understand the role of socio economic status to control the transmission of HIV/AIDS.

